# Virtuelle Realität in der Lehre im Fach Psychiatrie und Psychotherapie

**DOI:** 10.1007/s00115-021-01227-5

**Published:** 2021-11-04

**Authors:** Paraskevi Mavrogiorgou, Pierre Böhme, Vitalij Hooge, Thies Pfeiffer, Georg Juckel

**Affiliations:** 1 Klinik für Psychiatrie, Psychotherapie und Präventivmedizin, LWL-Universitätsklinikum, Ruhr Universität Bochum, Alexandrinenstr. 1, 44791 Bochum, Deutschland; 2 Raumtänzer GmbH, Nickelstr. 21, 33378 Rheda-Wiedenbrück, Deutschland

**Keywords:** Virtuelle Realität, Mixed reality, Lehre, Psychiatrie, Avatare, Virtual reality, Mixed reality, Education, Psychiatry, Avatar

## Abstract

**Hintergrund:**

Ausbildung und Lehre müssen sich den Gegebenheiten insbesondere in Corona-Zeiten anpassen, zumal neue digitale Technologien zur Verfügung stehen. Ärztliche Interaktions- und Explorationstechniken sind die wichtigsten Werkzeuge, die Medizinstudierende im Fach Psychiatrie und Psychotherapie zu erwerben haben.

**Ziel der Arbeit:**

Avatare in virtueller Realität (VR) können grundsätzlich alle Krankheitsbilder in unterschiedlichen Schweregraden zu jeder Zeit repräsentieren.

**Material und Methoden:**

Im Bochumer Avatar-Explorationsprojekt (AVEX) treten Studierende in den Dialog mit „psychisch kranken“ Avataren und versuchen, unter Anleitung und Supervision Diagnose, Differenzialdiagnose und Behandlungsempfehlungen zu erarbeiten.

**Ergebnisse und Diskussion:**

Dadurch können die Studierenden auch seltene oder schwere psychiatrische Krankheitsbilder durch VR vermittelt kennenlernen. Dieser Übersichtsartikel stellt erste Erfahrungen insbesondere in Aufbau und Entwicklung sowie bez. der technologischen Herausforderungen dar.

## Abstract (en)

**Background:**

Since the COVID-19 pandemic medical training and teaching have to adapt to the new circumstances, especially as new digital technologies become available. Physician’s interaction and exploration techniques are among the most important tools that medical students have to acquire in psychiatry and psychotherapy.

**Objective:**

Virtual reality (VR) avatars can basically represent all syndromes in varying degrees of severity at any time.

**Material and methods:**

In Bochum’s avatar exploration project (AVEX), students enter into dialogues with “mentally ill” avatars and, under guidance and supervision, try to work out the diagnosis, differential diagnoses and treatment recommendations.

**Results and discussion:**

This allows students to learn about rare or severe psychiatric conditions presented in VR. This review article presents first experiences especially in setting up and development as well as regarding the technological challenges.

## Hintergrund

Die seit nunmehr über einem Jahr die Menschheit geißelnde Corona-Pandemie scheint den digitalen und medientechnischen Fortschritt in Windeseile zu beflügeln. Der Einsatz und Gebrauch neuer Medien vereinfacht unser tägliches Miteinander, zumal das Virus eine unsichtbare und nicht minder lebensgefährliche Barriere zwischen uns schafft. Vor allem im Kontext unserer Arbeit mit Menschen, die Hilfe bedürfen, und dem dazu überaus notwendigen kollegialen Austausch von Informationen stellen die technischen Möglichkeiten im Sinne einer „positiven Technologie“ gerade in dieser Zeit der sozialen Distanzhaltung einen immensen Benefit dar [18]. Dies wiederum motiviert, die bereits vorhandenen Technologien noch effektiver zu nutzen und weiter in der Entwicklung voranzutreiben. Vor allem im Bereich der Medizin, hier speziell der Psychiatrie und Psychotherapie, spielt die Nutzung von Technik und Medien, wie z. B. die verschiedenen videotechnologischen Methoden zur Aufnahme und Darstellung psychopathologischer Verhaltensänderungen, aber auch zu Lehrzwecken, schon lange eine große Rolle [5]. Daher verwundert es nicht, dass in diesem Bereich schon seit einer Reihe von Jahren auch neue technische Verfahren, wie die VR(„virtual reality“)-Technologie, zur Diagnostik und Therapie psychischer Störungen genutzt werden [2, 17]. Trotzdem muss man einschränkend festhalten, dass bis dato die VR-Technologie sowohl klinisch, wissenschaftlich und als auch als Lehr- und Lernmethode für Studierende der Medizin sowie Facharztkandidaten keine flächendeckende oder gar etablierte Vorgehensweise darstellt [13, 14]. In der folgenden Arbeit soll daher die VR-Technologie hinsichtlich ihrer bisherigen und zukünftigen Einsatzmöglichkeiten vor allem im Fachgebiet von Psychiatrie und Psychotherapie am Beispiel des Bochumer Avatar-Explorationsprojektes („AVEX“) als eine nützliche Möglichkeit in der Lehre von Medizinstudierenden, aber auch in der Fort- und Weiterbildung von Weiterbildungskandidaten und sonst in der Psychiatrie Tätigen näher dargestellt werden.

## VR-Methodik und Terminologie

Unter dem allgemeinen Begriff virtuelle Realität (VR) werden Erfahrungsräume verstanden, in die Menschen eintauchen können, die jedoch vollständig synthetisch sind und meistens durch einen Computer generiert werden, also digitaler Natur sind.

**Figure 1:**
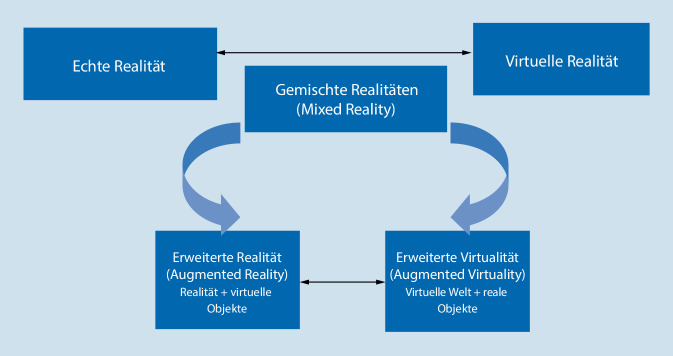

Die weiteren Begriffe „mixed reality“ bzw. „augmented reality“, die in dem Kontext teilweise auch synonym erwähnt werden, bezeichnen unterschiedliche Ausprägungen entsprechend dem Realitäts-Virtualitäts-Kontinuum-Modell von Milgram und Kishino ([15]; Abb. 1). Milgram und Kishino [15] gehen vom Oberbegriff „mixed reality“ (MR; gemischte Realität) aus und verstehen darunter die Kombination von realen und digital erzeugten (virtuellen) Objekten. Je nach Kombination bzw. Durchmischungsgrad ist ein kontinuierlicher Übergang von echter Realität über die „augmented reality“ (AR; erweiterte Realität mittels Ergänzung virtueller Elemente wie z. B. 3‑D-Animationen über Smartphones), des Weiteren der „augmented virtuality“ (AV; erweiterte Virtualität durch Hinzufügen realer Elemente wie Personen in die virtuelle Welt, wie z. B. Computerspiele, in denen der Nutzer in Echtzeit agiert) bis hin zur vollständigen virtuellen Realität (VR) möglich, bei der die reale Welt komplett ausgeblendet wird und der Nutzer, z. B. mittels einer VR-Brille, ganz in einer virtuellen Realität „eintaucht“.

In einer so digital erzeugten „künstlichen Welt“, ob sie „imaginär, symbolisch“ ist oder „gewisse Aspekte der realen Welt simuliert“ [20], soll dem Nutzer die Möglichkeit gegeben werden „eine sensomotorische und kognitive Aktivität aufzubauen“, die der in realen Situationen entspricht. Nach Roy und Schlemminger [20] ist das als das wesentliche Ziel der VR zu sehen und nicht eine „virtuelle Welt zu erschaffen, die so realitätsgetreu wie möglich ist“. Um das Ziel bestmöglich im Sinne realer bzw. nichtkünstlicher Wahrnehmungserlebnisse des Nutzers zu erreichen, sind neben den subjektiven Faktoren des Nutzers u. a. zwei wesentliche technisch determinierte Voraussetzungen von Bedeutung:

Zum einen die sog. *Immersion* [21], die als ein Maß der Stärke der Eingebundenheit des Nutzers in die virtuelle Umgebung verstanden wird. Die Immersion, die unabhängig von subjektiven Aspekten und Erfahrungen der Nutzer ist, ist umso größer, je mehr und je stärker die verschiedenen Wahrnehmungssinne der Nutzer allein durch die technischen Gegebenheiten angeregt werden. Nach Barbe et al. [2] ist daher die Immersion kein psychologisches Merkmal, sondern eine Kenngröße der eingesetzten VR-Technologie, die, z. B. je nachdem, welche Darstellungsform (unterschiedliche Sichtfelder von Monitoren vs. VR-Brille) oder ob haptische Geräte verwendet werden, in die eine oder andere Richtung beeinflusst werden kann.

Der zweite Faktor betrifft die *Interaktion* zwischen Mensch und Computer und umfasst die technischen Möglichkeiten, die es dem Nutzer ermöglichen, verbal (z. B. Spracherkennung) oder nonverbal (Bewegungserfassung, z. B. der Finger mittels Tracking) mit der künstlichen Welt bzw. virtuellen Umgebung zu interagieren [20].

Je überzeugender eine virtuelle Welt dem Nutzer durch die Technik vermittelt wird, desto wahrscheinlicher ist es, dass in ihm ein sog. Präsenzgefühl entsteht. Hierbei bezeichnet *Präsenz* das Gefühl des Nutzers, in die virtuelle Welt eingebunden zu sein [21]. Präsenz kann somit als das subjektive Erlebnis des Nutzers, als eine emotional-kognitive psychophysiologische Reaktion, und nach Roy und Schlemmiger [20] auch als eine „Wahrnehmungsillusion“ verstanden werden, da man die dahintersteckende VR-Technik („Mediatisierung“) nicht mehr wahrnimmt.

Gerade in Bezug auf das Präsenzgefühl als eine subjektive Kenngröße, die durch zahlreiche individuelle Eigenschaften des Nutzers, wie z. B. die Bereitschaft, sich auf die virtuelle Realität einzulassen, aber auch seine diesbezüglichen Erwartungen, erscheint eine weitere Differenzierung, wie bei Roy und Schlemmiger [20] vorgenommen, sinnvoll. Hierbei wird zwischen der räumlichen (VR-Objekte werden als real erlebt), der sozialen (VR-Figuren sog. „Avatare“ vermitteln das Gefühl realer sozialer Interaktion), der Selbstpräsenz (Nutzer hat eine Vorstellung von sich selbst in der virtuellen Realität) und der Handlungspräsenz (Handeln des Nutzers hat einen ihm bewussten veränderten Einfluss auf die virtuelle Realität) unterschieden [20]. Nach Barbe et al. [2] scheint das Erzeugen eines Präsenzgefühls und vor allem sozialer Präsenz wesentlich zu sein, um beim Nutzer in einer immersiven virtuellen Realität psychophysiologische Reaktionen auszulösen, die denen in realen Situationen entsprechen. Darin begründen sich auch der Nutzen und die Vorteile der VR-Technologien, die es ermöglichen, reale Situationen in einer virtuellen Welt zu implementieren und zu simulieren sowie dabei beim Nutzer ein ihm vertrautes, sich real anfühlendes Reaktionsmuster auslösen. Dies wird insbesondere im Einsatz der VR-Anwendungen in den verhaltenstherapeutisch orientierten Expositionsverfahren deutlich, auf die weiter unten noch eingegangen wird.

## Klinisch-therapeutischer Einsatz der VR-Technologie

Seit den ersten Einsätzen der VR-Anwendungen v. a. zur Behandlung von Ängsten bzw. Phobien im Zusammenhang mit einer zahnärztlichen Behandlung bereits Ende der 1980er-Jahre (z. B. [16]) konnte durch die stetige Weiterentwicklung der technischen Möglichkeiten VR auch in verschiedenen medizinisch-klinischen Anwendungsgebieten [8–10], vorzugsweise hier als Lernmethode, ausgeweitet werden. Aus Kapazitätsgründen müssen hier die teilweise spannenden und innovativen Studien und Berichte (eine aktuelle Übersicht dazu in Yeung et al. [23]) unberücksichtigt bleiben, zumal auch die Literatur zum Einsatz der VR-Anwendungen im Bereich von Psychiatrie und Psychotherapie in den letzten 10 bis 15 Jahren massiv angestiegen ist. Das zeigt sich auch darin, dass mittlerweile mehrere Übersichtsarbeiten und zuletzt auch Metaanalysen veröffentlicht wurden (z. B. [3, 12, 19]). So identifizierten Cieslik et al. [3] im Rahmen ihrer Recherchen in PubMed und Web of Science nahezu 850 Arbeiten zur Übersicht von VR-Technologien und psychischen Störungen, wobei letztlich nur 70 als Übersichtsartikel in die finale Analyse eingeschlossen werden konnten. Die zusammenfassenden Ergebnisse hierbei zeigen den effektiven Einsatz von VR-Anwendungen bei der Behandlung verschiedenartiger Schmerzsyndrome, in dem Schmerzpatienten mittels VR-Brillen z. B. in für sie angenehme, entspannungsfördernde virtuelle Umgebungen eintauchen oder durch immersive Animationsspiele abgelenkt werden. Ebenso gut ist auch die Wirksamkeit der VR-Anwendungen bei der Behandlung spezifischer Phobien und Angstzustände belegt, wobei hier in Analogie zu den verhaltenstherapeutischen Expositionsverfahren in vivo die betroffenen Patienten mittels digitaler Simulation mit den angstbesetzten Stimuli (Objekte und/oder Situationen) konfrontiert werden und sie in der jeweiligen virtuellen Umgebung dann entsprechend psychopathologisch reagieren. Weniger gut gesichert, da insgesamt auch weniger bis dato untersucht, ist die Wirksamkeit der VR-Technologie bei der Behandlung schwerer endogener psychischer Störungen wie z. B. den affektiven Störungen, Erkrankungen aus dem schizophrenen Formenkreis, der Demenzen oder auch bei Substanzabhängigkeit. Zurzeit gibt es zwei Cochrane-Metaanalysen zu diesem Themenbereich, einmal zur Behandlung schizophrener Patienten mit akustischen Halluzinationen durch VR-Methoden und einmal über weitere schwere psychiatrische Störungsbilder wie affektive und psychotische Störungen [1, 7]. Beide kommen angesichts der derzeit noch nicht ausreichendenden Datenlage zu dem Schluss, dass die VR-Technologie ein interessanter Behandlungsansatz für psychische Störungen insbesondere in der Verbesserung bestimmter kognitiver, emotionaler und sozialer Funktionen darstellen würde, aber noch längst nicht ausreichend validiert sei. Hier sehen Cieslik et al. [3] einen weiteren Forschungsbedarf und die Notwendigkeit der Durchführung randomisiert-kontrollierter Studien (RCTs).

## Einsatz als diagnostisches Instrument

Auch in Bezug auf den Einsatz von VR-Anwendungen zur Diagnosestellung psychischer Erkrankungen finden sich bereits einige vielversprechende Berichte. So fassen Clay et al. [4] in ihrer auf insgesamt 9 Studien basierenden Übersicht zusammen, dass immersive VR-Anwendungen hilfreich bei der Identifikation und Differenzierung von Patienten mit einer Alzheimer-Erkrankung und Patienten mit leichter kognitiver Beeinträchtigung sein können.

Washington et al. [22] sehen in der digitalen Transformation und Implementierung der gegenwärtigen diagnostischen Klassifikationssysteme eine deutlich bessere Möglichkeit, Erkrankungen aus dem Autismusspektrum frühzeitig zu identifizieren. Diesbezüglich ist es bereits möglich, anhand der bestehenden VR-Technologie eine Differenzierung autistisch erkrankter Kinder von Kindern mit anderen neurologischen Erkrankungen vorzunehmen [22]. Interessant erscheint auch der von Barbe et al. [2] dargestellte Einsatz der VR-Anwendungen in der forensischen Psychiatrie zur Diagnostik sexueller Deviationen mittels digitaler Präsentation entsprechender Stimuli oder computergenerierter Avatare. Dennoch bedarf es hier weiterer Bemühungen, die technischen Möglichkeiten von VR weiterhin auch inhaltlich anzupassen und zu optimieren, sodass zunehmend ihr Einsatz als ergänzendes diagnostisches Instrumentarium im Bereich der Psychiatrie und Psychotherapie ausgebaut werden könnte. Dabei darf, wie Barbe et al. [2] treffend formulieren, der hierzu notwendige technische wie auch konzeptuell-inhaltliche Aufwand nicht unterschätzt werden.

## Einsatz als Lern- und Lehrmethode

Die Corona-Pandemie und die damit verbundenen gesellschaftlichen Folgen haben vor allem zur Demaskierung des Nichtvorhandenseins digital-technischer Lern- und Lehrangebote nicht nur in den Schulen, sondern auch in der medizinischen Ausbildung der Universitäten beigetragen. Gleichzeitig führte dies zur Notwendigkeit einer raschen, stärkeren Implementierung und Anwendung der schon bereits bestehenden Technologien im Rahmen des Medizinstudiums [14]. Bereits vor der Pandemie haben Kuhn et al. [13] im Kontext ihrer Übersichtsarbeit zu digitalen Lehr- und Lernangeboten in der medizinischen Ausbildung auf die Bedeutung der Digitalisierung hingewiesen und die Empfehlung formuliert, diese als kurrikulären Inhalt im Medizinstudium zu verankern.

Hinsichtlich der VR-Anwendungen als Lern- und Lehrmethoden in der Medizin finden sich in stetig steigender Zahl positive Berichte vor allem in Form digitaler Simulationen (z. B. anatomische Sektionen) oder der Verwendung virtueller Patienten (=Avatare; [6, 11, 13]). Computerbasierte Simulationen und VR-Anwendungen mit Avataren haben nach Piot et al. [17] nach ihrer ersten Sichtung von 46.571 Studien und der finalen Analyse von 163 Studien (von denen nur 27 RCTs waren) einen positiven Lerneffekt bei der psychiatrisch-psychotherapeutischen Ausbildung von Medizinstudierenden, aber auch psychiatrischer Professioneller. Die Autoren fassen jedoch zusammen, dass trotz der Vielzahl der Veröffentlichungen vor allem in den letzten 10 Jahren und aufgrund der Heterogenität der Studien nicht davon ausgegangen werden kann, dass der Einsatz digitaler Technologie in der psychiatrisch-psychotherapeutischen Ausbildung als flächendeckend etabliert angesehen werden darf, und hier ein weiterer Optimierungsbedarf besteht [17].

## Bochumer AVEX-Projekt mit „psychisch kranken“ Avataren

Lange vor Corona wurde an der Klinik für Psychiatrie, Psychotherapie und Präventivmedizin des LWL-Universitätsklinikums der Ruhr-Universität Bochum mit Fördermittel des Landes NRW für die medizinische Lehre begonnen, „psychisch kranke“ Avatare zu erarbeiten und digital zu hinterlegen. Da neue Medien und Technologien eine immer größere Rolle in der heutigen Gesellschaft, im Berufsleben und speziell in der Medizin spielen, jedoch digitale Kompetenzen im Medizinstudium bislang nur wenig vermittelt werden, bestand das Ziel hier darin, für das Fach Psychiatrie und Psychotherapie digitale Anwendungen in der VR und telemedizinische Strukturen, sprich das Diagnostizieren und Behandeln elektronisch aus der Ferne, als „Medizin der Zukunft“ in die Lehre zu integrieren und entsprechende Fertigkeiten zu vermitteln. Dabei sollten spezifische Fertigkeiten für dieses Fach durch Simulation in der virtuellen Realität mit der grundsätzlichen Möglichkeit der nichtbegrenzten, zeitlich simultan vorhandenen psychiatrischen Fälle und ihrer diagnostischen und/oder therapeutischen Komplexität besser erworben und trainiert werden können. Das Training explorativer und diagnostischer Fähigkeiten könnte somit deutlich leichter, unproblematischer und gesteuert nach Intensität und Komplexität in der virtuellen Realität mittels programmierter Avatare als „Träger“ bestimmter Krankheitskonstellationen und bestimmter klinischer Situationen erfolgen. Gleichzeitig könnte durch digitale Aufzeichnung dem Übenden ein großes Set von Rückmeldungen (Fragestrategie, Wortwahl, Körperhaltung usw.) einschließlich Biometrie (z. B. HF, RR, Hautwiderstand usw.) gegeben werden, die zur weiteren Optimierung seiner Lern- und Explorationsstrategien genutzt werden würden. Darüber hinaus könnten zum einen Aspekte der ärztlichen Interaktion mit psychiatrisch schwierigen Patienten simuliert und geübt werden und zum anderen alle beliebigen, aber auch seltene Patientenfälle, sodass den Medizinstudierenden Kenntnisse und Fähigkeiten in der Exploration, Erhebung des psychopathologischen Befundes und Diagnosestellung (und ggf. Behandlungsvorschläge) aller psychiatrischen Erkrankungen (ICD 10: F0-9) ermöglicht werden würden. Auch wenn dies nur eine gewisse Annäherung an die „echte“ klinische Situation und Patienten bedeutet, entkoppelt dieser Ansatz den Studentenunterricht ein stückweit von den aktuellen Patientenaufnahmen, sodass Krankheitsbilder kennengelernt werden können, die sonst selten in den Kliniken zu finden sind oder bei denen die Patienten so schwer krank sind, dass sie am Unterricht nicht teilnehmen können oder wollen. Zudem würden anders als bisher, neuropsychologische Testverfahren, Persönlichkeitsdiagnostik und ggf. auch basale psychotherapeutische Strategien mit integriert, simulierend gelernt und geübt werden können. Studierende sollen also zusammenfassend bei AVEX Folgendes lernen: (1) das Explorieren von und Üben mit Krankengeschichten, Befunde und Behandlungskonstellationen „tragenden“ Avataren; (2) die Einschätzung ihres eigenen Tuns durch Rückmeldung analysierter Daten von ihnen selbst (Sätze, Worte usw. sowie Biometrie) und (3) die Durchführung diagnostischer und psychotherapeutischer Gespräche in der VR zum Training insbesondere verbaler Behandlungsformen mit anschließender Erfolgskontrolle.

**Figure 2:**
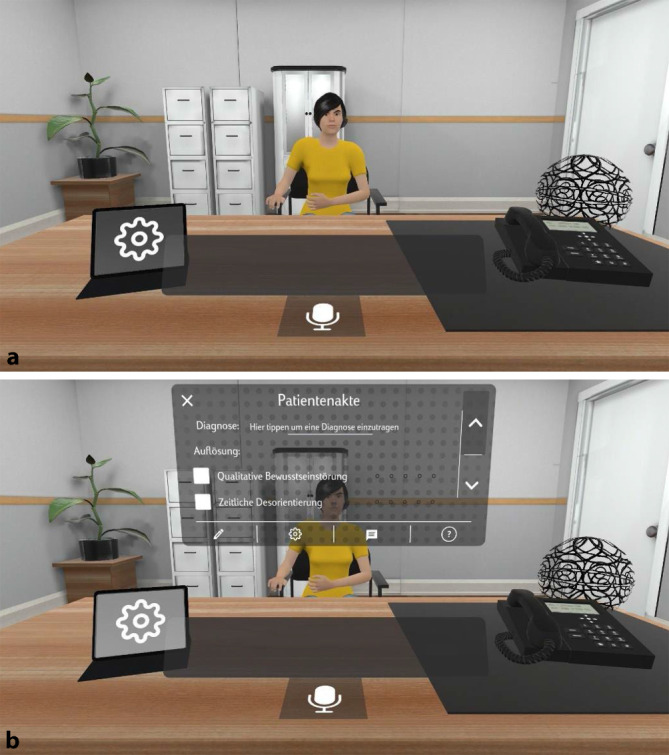

Für die konzeptuelle Entwicklung der psychisch kranken Avatare war es zunächst erforderlich, diese sowohl psychopathologisch (gemäß AMDP) als auch diagnostisch entsprechend den aktuellen diagnostischen Klassifikationssystemen (ICD-10, DSM‑5 und ICD-11) zu skizzieren und im weiteren Schritt diese Daten in das von den Informatikern eigens entwickelte Flux-Trainings-Management-System (FluxTMX) zu implementieren. Dieses Programm ermöglicht darüber hinaus, verschiedene Avatare (= Charaktere) neben einem Krankheitsprofil jeweils mit einer individuellen Biografie, einem eigenen Aussehen und individueller Motorik auszugestalten (Abb. 2). Des Weiteren erfolgte das Einpflegen von Fragen, die im Rahmen einer psychiatrisch-psychotherapeutischen Exploration zur Biografie, Krankheitsanamnese bezüglich psychischer, aber auch somatischer Erkrankungen, Medikamenten- und Drogenanamnese, Familienanamnese sowie zur aktuellen Symptomatik und Psychopathologie üblicherweise gestellt werden. Analog dazu wurden entsprechende allgemeine, aber auch je nach Avatar individuell spezifische Antworten eingegeben und mit den entsprechenden Fragen mittels Wahrscheinlichkeitsarithmetik und Schlüsselwörtern gekoppelt.

Die Kernidee des Ansatzes ist es dabei, dass die Avatare durch ein Krankheitsprofil individuell konfiguriert werden können und sich die Antworten auf viele Fragen dann automatisch aus dem Profil über ein Wahrscheinlichkeitsmodell ableiten lassen. So muss der Lehrende nur noch das Profil umkonfigurieren, um einen neuen, interessanten Avatar-Patienten zu modellieren.

Die momentane Version erlaubt es dem Nutzer, in einer immersiven virtuellen Explorationssituation den Avatar-Patienten bez. biografischer und krankheitsbezogener Aspekte zu befragen und einen nosologiespezifischen psychopathologischen Befund zu erheben, sodass eine diagnostische Einschätzung erfolgen kann. Am Ende der Exploration/Trainingseinheit erfolgt digital der Abgleich mit dem hinterlegten Befund und der Diagnose, sodass der Nutzer unmittelbar ein Feedback erhält (Abb. 2).

Das FluxTMS ist dabei mehrbenutzerfähig, sodass mehrere Trainingssimulationen mit verschieden konfigurierbaren Avataren parallel durchgeführt und umfangreich protokolliert werden können. So einfach wie auch plausibel sich diese Projektbeschreibung liest, so überraschender war die Erkenntnis, dass trotz technischer Errungenschaften die Realisierung weitaus aufwendiger sowohl personell, finanziell als auch zeitlich ausfiel, als ursprünglich angenommen. Neben der Spracherkennung und Sprachproduktion bestand die hauptsächliche Problematik darin, ein flexibles, aber gleichzeitig spezifisches Explorationsprogramm zu entwickeln, einerseits basierend auf den von den Informatikern hinterlegten Wahrscheinlichkeitsalgorithmen, andererseits die alltägliche psychiatrische Realität bestmöglich abbildend. Die Schwierigkeit der Zusammenführung dieser beiden unterschiedlichen, aber jeweils notwendigen Ziele zeigt sich im folgenden konkreten Beispiel. Unsere weibliche Avatarin Lara B. (geb. am 04.07.1994) mit der Diagnose einer paranoiden Schizophrenie leidet neben einem systematisierten Beziehungs- und Beeinträchtigungswahn unter dialogisierenden und kommentierenden Stimmen. Dieses psychopathologische Merkmal findet sich jedoch nicht bei unserem Avatar Paul. W (geb. 17.08.1959), der unter einer rezidivierenden depressiven Störung, gegenwärtig schwere Episode ohne psychotische Symptome, leidet. Auf die Frage „Hören Sie fremde Stimmen, obwohl keiner in Ihrer Nähe ist, und fühlen Sie sich von diesen gesteuert?“ bestand nun aufgrund des Wahrscheinlichkeitsalgorithmus anfänglich die Problematik, dass sowohl Lara B. als auch Paul W. mit jeweils 50 %iger Wahrscheinlichkeit die nicht für sie nosologiespezifische falsche Antwort wählten. Die durch die hinterlegten auf Wahrscheinlichkeit basierenden Algorithmen bedingte Flexibilität musste für einen Teil der psychopathologischen Merkmale, die richtungsweisend für eine psychiatrische Diagnose sind, aufgegeben werden. Flexibilität, aber auch eine „spezifische“ Individualität sowohl auf der Seite des Nutzers als auch auf der der Avatare scheinen durch die mögliche Durchmischung und Kombination eine große Herausforderung zu sein, der man zukünftig jedoch zunehmend besser gerecht werden muss. Bis jetzt sind Avatare aller psychiatrischen Hauptkrankheitsbilder entstanden, die gegenwärtig bez. ihrer Tauglichkeit für den Studentenunterricht geprüft, optimiert und evaluiert werden. Die größte Herausforderung ist hierbei, dass ein flüssiger Dialog entsteht. Zum funktionierenden Dialog braucht es daher eine sehr gut funktionierende und schnelle Spracherkennung, damit der Computer im Hintergrund die Fragen der Untersuchenden versteht und die passenden Antworten ausgeben kann. Um die Avatare auch körperlich überzeugend darstellen zu können, sind diese nach der Programmierung des logischen Grundgerüsts aufwendig visualisiert worden. Je nachdem, welche psychischen Probleme die Avatare repräsentieren, soll dies auch durch ihre Körpersprache repräsentiert werden. Sie können im Gespräch sitzen, stehen, liegen oder herumlaufen. Auch ihre Mimik soll dem geübten Beobachter Aufschluss über die zugrunde liegende Erkrankung geben können. Den Studierenden soll vor allem nicht langweilig werden. Angesicht der Welten, die im Freizeitbereich die Spielindustrie bietet, muss das Angebot für die Studierenden anspruchsvoll sein und die Neugier auf die Begegnung mit den virtuellen Patienten wecken. Dafür sind die Avatare modular aufgebaut worden, sodass sich der Schwierigkeitsgrad der Exploration variieren lässt. Der ausgewählte Avatar stellt eine bestimmte psychiatrische Diagnose oder Behandlungssituation dar. Wie die Studierenden aber dahin kommen, welche Fragen sie genau stellen, ist nicht festgelegt, das ist das, was sie dann unter Anleitung und Supervision zu lernen haben.

## Schlussfolgerungen und Perspektiven

Virtuelle Realität hat sich in der Medizin durchgesetzt. Sie ist daher für den klinischen Einsatz im Rahmen von Psychiatrie und Psychotherapie relevant und auch für Fort- und Weiterbildung sowie Lehre interessant. Das Bochumer AVEX-Projekt zeigt erste wichtige Erkenntnisse für Aufbau und Nutzung solcher VR-Lernprogramme auf. Insbesondere die modulare Anlage von AVEX bez. Inhalten und Schwierigkeitsgraden ist didaktisch wertvoll und für den Studenten, wie erste Erfahrungen zeigen, anregend und lehrreich. Ziel wird es sein, dass insbesondere im Bereich der auch Corona-bedingten digitalen Lehre der Unterricht in der virtuellen Realität regelmäßig stattfindet und im VR-Labor, aber auch von zuhause den Studierenden erlauben wird, Patienten mit verschiedenen, aber auch schweren Krankheitsbildern kennenzulernen und ihre Explorationstechniken zu üben, die sonst im normalen Unterricht nur selten oder auch gar nicht mitwirken.

## Fazit für die Praxis

- Der Einsatz von VR und Avataren, die psychiatrische Krankheiten und Fragestellungen repräsentieren, erweitern und verbessern Lehre.
- Nicht nur in Pandemiezeiten können hierdurch eher seltene und schwergradige Störungsbilder kennengelernt und vertieft werden.
- Der Einsatz von VR fördert das Üben von Explorationstechniken im Rahmen der ärztlichen Interaktion bei allen Medizinstudierenden.
- Hierdurch wird der zentrale Stellenwert des Faches Psychiatrie und Psychotherapie in der ärztlichen Ausbildung gefördert.
